# Molecular development of fibular reduction in birds and its evolution from dinosaurs

**DOI:** 10.1111/evo.12882

**Published:** 2016-03-04

**Authors:** João Francisco Botelho, Daniel Smith‐Paredes, Sergio Soto‐Acuña, Jingmai O'Connor, Verónica Palma, Alexander O. Vargas

**Affiliations:** ^1^ Laboratorio de Ontogenia y Filogenia, Departamento de BiologíaFacultad de Ciencias de la Universidad de ChileSantiagoChile; ^2^ Área de PaleontologíaMuseo Nacional de Historia NaturalSantiagoChile; ^3^ Institute of Vertebrate Paleontology and PaleoanthropologyChinese Academy of ScienceBeijingChina; ^4^ FONDAP Center for Genomic Regulation, Departamento de BiologíaFacultad de Ciencias de la Universidad de ChileSantiagoChile

**Keywords:** Bird–dinosaur transition, fibula, IHH, mesozoic birds, PTHrP, zeugopod

## Abstract

Birds have a distally reduced, splinter‐like fibula that is shorter than the tibia. In embryonic development, both skeletal elements start out with similar lengths. We examined molecular markers of cartilage differentiation in chicken embryos. We found that the distal end of the fibula expresses Indian hedgehog (IHH), undergoing terminal cartilage differentiation, and almost no Parathyroid‐related protein (PTHrP), which is required to develop a proliferative growth plate (epiphysis). Reduction of the distal fibula may be influenced earlier by its close contact with the nearby fibulare, which strongly expresses PTHrP. The epiphysis‐like fibulare however then separates from the fibula, which fails to maintain a distal growth plate, and fibular reduction ensues. Experimental downregulation of IHH signaling at a postmorphogenetic stage led to a tibia and fibula of equal length: The fibula is longer than in controls and fused to the fibulare, whereas the tibia is shorter and bent. We propose that the presence of a distal fibular epiphysis may constrain greater growth in the tibia. Accordingly, many Mesozoic birds show a fibula that has lost its distal epiphysis, but remains almost as long as the tibia, suggesting that loss of the fibulare preceded and allowed subsequent evolution of great fibulo–tibial disparity.

The shank (zeugopod) of most tetrapods has two equally long bones—the medial (inner) tibia and the lateral (outer) fibula. In early theropod dinosaurs, which are bird ancestors, both bones were equally long, although the fibula is more narrow and in close contact to the tibia. This condition was still present in the basal bird *Archaeopteryx* (Ostrom 1973; Mayr et al. 2005). Within the Pygostylia, closer to modern birds, the fibula became shorter than the tibia and splinter‐like toward its distal end, no longer reaching the ankle (O'Connor et al. 2011a). In modern birds, the fibula is typically about two‐thirds the length of the tibia, but fibulo–tibial proportions show considerable evolutionary variation, with proportionally shorter or longer fibulae in different species (Owen 1866; Streicher and Muller 1992).

Distal fibular reduction occurs after early embryonic patterning of the limb skeleton, at postmorphogenetic stages: bird embryos start out with a fibula as long as the tibia, which contacts the ankle (Heilmann 1926). Fibular reduction can be avoided through experimental manipulations. Placing a barrier between the early cell populations that are precursors of the tibia and fibula generates late chicken embryos with a dinosaur‐like fibula, as long as the tibia (Hampe 1958, 1960aa,b; Müller 1989). Dinosaur‐like fibulae have also been obtained by adding more mesenchyme to the limb bud (Wolff and Hampe 1954), placing grafts of Sonic Hedgehog (SHH) expressing cells (also known as “ZPA cells,” Archer et al. 1983), and by misexpression of the *Hoxd‐13* gene in the entire early limb bud (Goff and Tabin 1997). A recurrent explanation is that there is a competition for cells between the two early precursor populations of the tibia and fibula, in which the tibia normally prevails. Adding more cells or separating early precursors with a barrier conceivably allows greater cell allocation to the fibula, which grows to a larger size. An alternative hypothesis is that fibular reduction results from the secondary loss of the growth plate at its distal epiphysis (Archer et al. 1983). A developmental program intrinsic to the fibula was proposed to make its distal end break off, with the resulting fragment becoming the fibulare (the heel bone, also known as the calcaneum, which is found distal to the fibula in all tetrapods). Without a growth plate, the distal fibula would then be incapable of growth. However, embryological descriptions of birds and other tetrapods show the fibulare initiates cartilage formation independently and at an earlier stage, rather than breaking off from the cartilaginous fibula (Shubin and Alberch 1986; Müller and Alberch 1990). It has also been noted that experimental manipulations have a stronger effect on the length of the tibia. As the increase of the fibula is moderate by comparison, this is considered the main effect leading to a dinosaur‐like phenotype, with a fibula as long as the tibia (Müller 1989; Goff and Tabin 1997). This has supported the view that fibular reduction is a side effect of competitive dominance and/or increased growth of the tibia, downplaying any role for the distal fibular epiphysis.

It is well known that lengthwise growth at the end of a long bone (the epiphysis) is possible as long as it retains a cartilaginous growth plate. Long bones across tetrapods (including crocodylians, bird's closest living relatives) follow a similar pattern of endochondral cartilage replacement by bone (Johnson 1933; Rieppel 1992, Rieppel 1993a,b,c, 1994; Reno et al. 2007; Mitgutsch et al. 2011; Diaz and Trainor 2015). Maturation of the chondrocytes and then ossification starts at the center of the cartilage (diaphysis) and expands toward both ends (epiphyses). Chondrocyte phenotypes change from rounded immature proliferating cells expressing collagen type 2a (Coll‐II) and collagen type 9 (Coll‐IX), to become flattened in parallel arrangements, and then terminally hypertrophic cells expressing collagen type 10 (Coll‐X). Lengthwise growth is allowed by the persistence of immature, proliferating chondrocytes at the ends of the epiphyses (growth plates; Kuhn et al. 1996; Wilsman et al. 1996; Farnum et al. 2007), and ceases when chondrocyte differentiation and ossification progress into them. This general pattern is conserved despite some differences among clades, such as the presence of secondary epiphyseal ossifications in mammals and lizards (Johnson 1933; Fabrezi et al. 2007).

Recently, important advances have been made in understanding the molecular mechanisms behind these processes (Crombrugghe et al. 2001; Kronenberg 2003; Kobayashi and Kronenberg 2014; Kozhemyakina et al. 2015). The secreted protein Indian hedgehog (IHH), produced by prehypertrophic chondrocytes, was discovered to play an important role in each aspect of endochondral ossification (Vortkamp et al. 1996; Kronenberg et al. 1997; Karp et al. 2000; Kobayashi et al. 2002; Long et al. 2004). It stimulates immature chondrocytes transition to flattened and then hypertrophic chondrocytes, favoring the replacement of cartilage by bone in the diaphysis. IHH also stimulates the production of Parathyroid hormone‐related protein (PTHrP) by periarticular chondrocytes, a secreted protein that delays the differentiation of immature chondrocytes in the epiphysis. PTHrP in turn inhibits the production of IHH, generating a negative feedback loop that maintains the coordinated differentiation of immature chondrocytes in the growth plate (Vortkamp et al. 1996; Kronenberg et al. 1997; Karp et al. 2000; see Fig. 4E). Genetic inactivation of PTHrP causes premature ossification and decreased growth of the skeleton (Karaplis et al. 1994; Lanske et al. 1996); Conversely, misexpression of PTHrP in the whole cartilage delays chondrocyte differentiation and impairs ossification (Weir et al. 1996). This PTHrP‐IHH feedback system has been comprehensively studied in both chicken and the mouse (e.g., Inada et al. 1999; Long et al. 2001a,b; Ueta et al. 2001; Mak et al. 2006, 2008; Ruiz‐Perez et al. 2007). The mouse, like any mammal, is an outgroup to chicken that phylogenetically is maximally distant within the amniotes. This suggests these molecular mechanisms are highly conserved, and can be expected to be present in other reptiles, including crocodylians, which are closer to birds (Reno et al. 2007).

To better understand the development of the fibula, we studied the timing of reduction and ossification in several orders of birds. In the chicken, we studied the molecular development of cartilage maturation and growth plate formation in the tibia and fibula. We also examined the onset of the IHH‐PTHrP feedback system in the fibula, including experimental downregulation of IHH signaling. We show that the distal epiphysis of the fibula presents an abnormal molecular profile when compared to other bones, and that a normal growth plate fails to be established. We also examined key specimens of Mesozoic birds documenting the earliest stages in the evolution of fibular reduction. The fossil evidence shows that disruption of the distal epiphysis was an early event, and may have played a key role for subsequent increases in fibular reduction.

## Materials and Methods

### SKELETAL STAINING

Fertilized eggs of *Gallus gallus* (Chicken, Galliformes), *Anas platyrhynchos* (Mallard duck, Anseriformes), and *Nothoprocta perdicaria* (Chilean tinamou, Tinamiformes) were purchased from local farms. Fertilized eggs of *Taeniopygia guttata* (Zebra finch, Passeriformes), *Columba livia* (Rock pigeon, Columbiformes), and *Melopsittacus undulatus* (Budgerigar, Psittaciformes) were obtained from birds kept at facilities of the Faculty of Science, University of Chile. Eggs were incubated at 37.5°C and 60% humidity. Embryos were anesthetized in ice and fixed in methanol for at least two days. For cartilage staining, embryos were left in a solution of 0.025% Alcian Blue diluted in 1:5 Ethanol/Acetic acid for two days and cleared with 0.5% KOH solution. For bone staining, embryos were fixed for one hour in 10% buffered formalin and stained for two hours in a solution of 0.05% Alizarin Red diluted in water with 0.5% of KOH. Stained cartilages were photographed and measured using Leica Application Suite software (*n* = 10 for each stage).

### WHOLE MOUNT IMMUNOFLUORESCENCE

Whole mount immunofluorescence follow the protocol described in Botelho et al. (2014a). Muscles of embryos older than HH31 were dissected. The following primary antibodies were employed: Anti‐Collagen type 2 (II‐II6B3s, DSHB, 1:20); Anti‐Collagen type 9 (2C2, DSHB, 1:20), Anti‐Collagen type 10 (X‐AC9s, DSHB, 1:10), anti‐Hedgehog (sc‐9024, Santa Cruz Biotechnology, 1:100).

### SECTION IMMUNOFLUORESCENCE

As anti‐PTHrP antigenicity is lost in Methanol fixed embryos, we used crosslink fixative and sectioning to detected PTHrP production. Chicken embryos were fixed in 4% PFA for two days at 4ºC. They were then washed in PBS and cryo‐protected in 30% sucrose. Forty‐micrometer‐thick sections were cut in a sliding microtome equipped with a freezing stage. After epitome retrieval in buffer Citrate, sections were incubated with the three primary antibodies over night at 4ºC: anti‐Collagen type 2 (II‐II6B3s, DSHB, 1:40), anti‐PTHrP (sc‐9680, Santa Cruz Biotechnology, 1:100), and anti‐Hedgehog (sc‐9024, Santa Cruz Biotechnology, 1:200). Sections were washed in PBST and incubated in the three secondary antibodies over night at 4ºC: anti‐mouse, anti‐rabbit, and anti‐goat made in donkey (Jackson ImmunoResearch). The same protocol was used to label mitotic nuclei with anti‐phosphohistone‐3 (06‐570, Upstate, 1:500). Sections were mounted in slides, coverslipped, and photographed in an Olympus fluorescent microscope.

### IN SITU HYBRIDIZATION

Probe for *PTC* was kindly donated by Professor Clifford Tabin from the University of Harvard. *SOX‐9* in situ probe was made using the follow primers (designed from http://www.ensembl.org/index.html): forward CGTCTCTGCCGGCTTTAC, backward CCTTCTTCAGGTCCGGGT. Embryos were fixed overnight with 4% paraformaldehyde (PFA), washed in buffer phosphate (PBS), dehydrated in a methanol series, and stored at –20°C. Whole mount in situ hybridization was carried out following the protocol described in Gallus Expression In Situ Hybridization Analysis (http://geisha.arizona.edu/geisha).

### PHARMACOLOGICAL TREATMENT

We delivered 12 μl of 3 mg/ml solution of Cyclopamine (LC Laboratories, Woburn, MA) in 45% 2‐hydropropyl‐*b*‐cyclodextrin (HBC; Sigma, St. Louis, MO) into the amniotic cavity of embryos at HH27 (*n* = 48). We confirmed the effectiveness of hedgehog downregulation in the zeugopod by observing a significant decrease in Ptc1, one of its downstream targets, in limbs of cyclopamine‐treated embryos (Fig. S1A). Embryos treated at HH29 presented different phenotypes (Fig. S1B).

### FOSSIL SPECIMENS

The material used for this study is housed at the Henan Geological Museum, Zhengzhou, China (*Sapeornis chaoyanensis* 41HIII0405); Institute of Vertebrate Paleontology and Paleoanthropology, Beijing (*Jeholornis prima* IVPP V13550); Tianyu Museum of Nature, Pingyi County, Shandong Province (*Eopengornis martini* STM24‐1); Fossil Research and Development Center, Third Geology and Mineral Resources Exploration Academy, Gansu Provincial Bureau of Geo‐Exploration and Mineral Development, Lanzhou (*Qiliania graffini*  FRDC‐05‐CM‐006); and Chinese Academy of Geological Sciences, Institute of Geology, in Beijing (*Gansus* CAGS‐IG‐04‐CM‐001).

## Results

### THE DEVELOPMENTAL TIMING OF FIBULAR REDUCTION IN BIRDS

**Figure 1:**
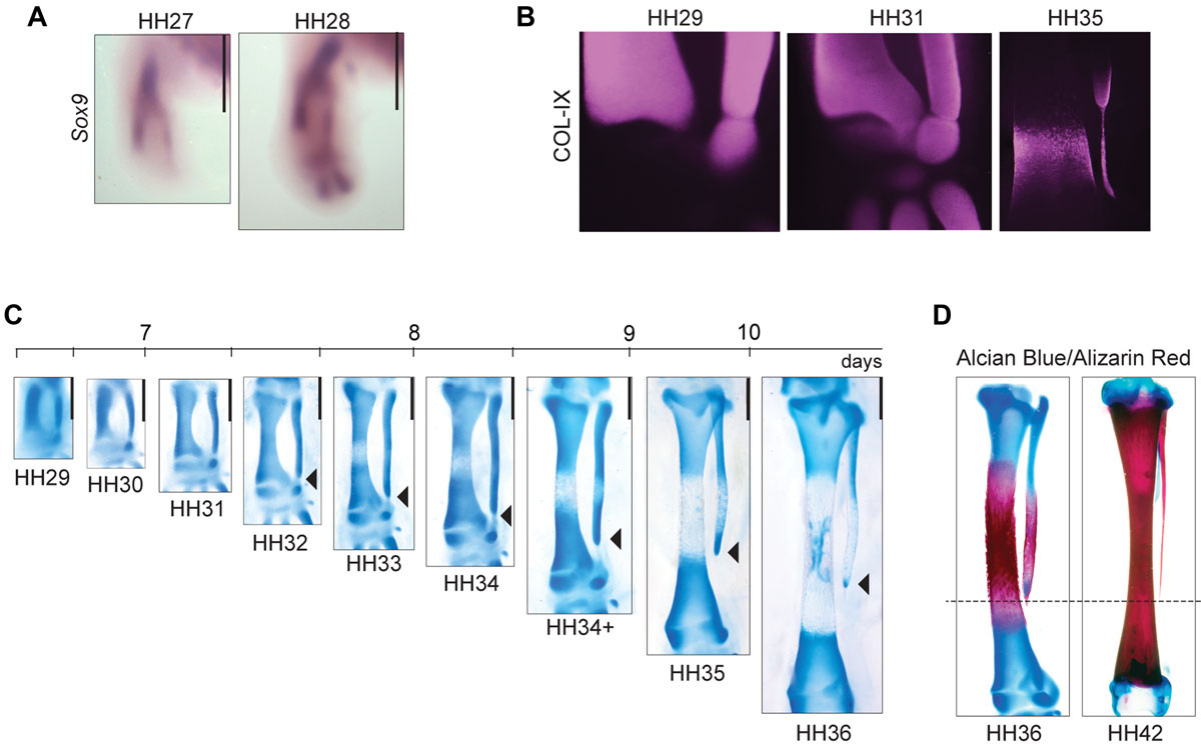
Avian fibular reduction occurs between HH31 and HH36. (A) *Sox9* in situ hybridization shows the earliest development of tibia and fibula; (B) Expression of Coll‐IX, a marker of early cartilage, shows the fibula is separate but in close contact with the fibulare during early development, later detaching from each other. (C) Early chicken hind limbs stained with Alcian blue reveal the growth and maturation of zeugopodial cartilages between embryonic days 6 and 10. Cartilage composes the entire element at earlier stages, while bone differentiation at the center is revealed by the absence of staining. Scale: 1 mm. (D) Double staining for cartilage and calcified bone at HH36 and HH42 shows that the length ratio of the tibia/fibula is established at HH36 and is maintained from that stage onward (not in scale).

We observed species from six different orders of birds, including the Chilean Tinamou, a member of Paleognathae. This lineage is phylogenetically maximally distant from the Neognathae, the other major lineage of modern birds that contains most species. In all the species observed, the embryonic tibia and fibula start out with the same length (see expression of the transcription factor *Sox9* at HH27 and HH28 in the chicken) and are still roughly equal as late as stage HH31 (Fig. 1). This is true even for species with a strongly reduced adult fibula, such as the budgerigar and the zebra finch (Fig. S2): postmorphogenetic reduction is common to all crown birds observed. We confirmed that in the chicken, the fibulare forms as a separate element, but then becomes closely appressed to the fibula, with a flattened contact surface between them (Fig. 1B, C). This condition is unlike other amniotes, in which a distance separates these bones (Fig. S3). The tibiale (the anklebone, found distal to the tibia) develops later than the fibulare, at HH31/32. The fibula only becomes conspicuously shorter than the tibia at HH33/34, when the distance between the distal fibula and the fibulare increases (Fig. 1B, C). The difference keeps increasing until HH36: by then, the fibulo–tibial ratio is roughly the same as in the adult (Fig. 1C, D). This is also true for other species, despite evolutionary variation among adult fibulo–tibial ratios (Fig. S4). Our data suggest that in all bird species, mechanisms of fibular reduction are at work between HH31 and HH36. In chicken, those stages correspond to the period between embryonic days 7 and 10, when length of the tibia increases in 5.61 mm (from 1.69 ± 0.14 mm at HH31 to 7.30 ± 0.18 mm at HH36) and the fibula only 2.52 mm (from 1.60 ± 0.09 mm at HH31 to 4.12 ± 0.13 mm at HH36). In all species, a bone collar occupies the center of the tibia at HH36, whereas the bone collar of the fibula is off‐center and distally displaced, almost reaching the distal end (Figs. 1C and S5). After HH36, the distal fibula becomes depleted from cartilage, growing exclusively by deposition of bone (Fig. 1D). Epiphyseal growth only in the proximal end generates the splinter‐like morphology of the distal end at later stages. This splinter‐like morphology is also observed in other bones that lose an epiphysis (e.g., the ulna in bats) or whose diaphysis is fractured at early development (e.g., the malleoare in several artiodactyls), as epiphyseal growth at only one side generates uneven thickness (Walmsley 1918; Sears et al. 2007). It is not the case for bones that lose one epiphysis at late development, or have relatively moderate growth (e.g., the phalanges and metatarsi of therian mammals; Reno et al. 2006).

### THE FIBULA FAILS TO DEVELOP A DISTAL GROWTH PLATE

**Figure 2:**
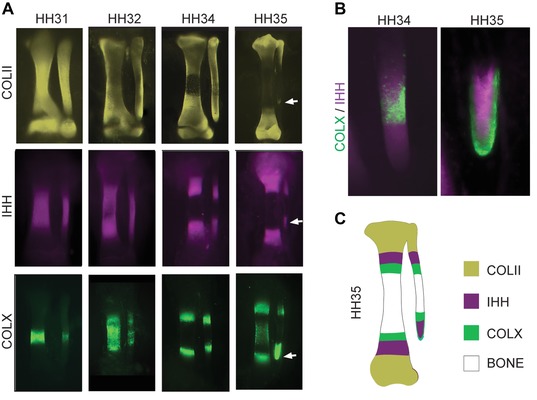
The distal fibula fails to develop its distal growth plate. (A) Immunofluorescence against Coll‐II, IHH, and Coll‐X proteins between HH31‐35 in the chicken embryo allows visualization of the dynamics of maturation and morphological change during skeletal morphogenesis. Not in scale. (B) Double staining of Coll‐X and IHH indicates the loss of distal fibular growth plate at HH35. (C) Schematic representation of the domains of expression of Coll‐II, IHH, and Coll‐X in the developing zeugopodial bones at HH35.

To understand the development of the tibia and fibula between HH31 and HH36, we used a whole mount immunofluorescence protocol to observe the expression of molecular markers of cartilage maturation (Botelho et al. 2014a). Initially, Coll‐II, a marker of immature chondrocytes, is expressed in the entire tibia and fibula. Maturation begins at midlength of the tibia, where Coll‐II ceases to be expressed. As maturation progresses, the Coll‐II‐free region expands, but does not reach the ends, where cell proliferation and lengthwise growth of the tibia occurs. In the fibula, however, the Coll‐II‐free region progresses almost immediately down to its distal end (Fig. 2A, white arrow), whereas at the proximal end, Coll‐II continues to be strongly expressed.

**Figure 3:**
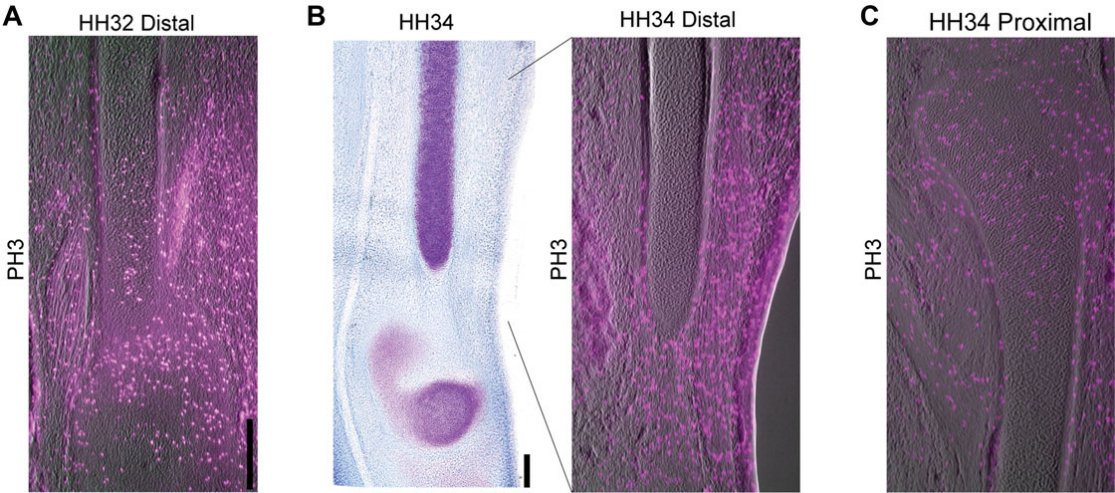
Cell proliferation ceases in the distal fibula. (A) Phosphohistone‐H3 staining shows a population of proliferating cells in the distal fibula at HH32. (B) The distal end of the fibula does not show proliferating cells, whereas the proximal zone conserves a population of chondroblasts actively proliferating at HH34. Scale: 100 μm.

IHH production, which indicates the onset of chondrocyte differentiation, also begins at midlength of the tibia and then separates into two distinct bands of expression, each progressing as a wave toward each extreme (Fig. 2A). However, they do not progress into the very end, presumably because of negative regulation of IHH by sustained PTHrP expression at the epiphyses. In the fibula, the proximal end conforms to this same pattern, but toward distal, the band of IHH progresses much farther, and eventually, all the way down to the tip of the element (Fig. 2A, B, white arrow). The expression of COll‐X, an indicator of hypertrophy and cartilage maturation, behaves like IHH: a wave‐like band of expression stops at a short distance from the ends of the tibia and of the proximal fibula, but progresses into the distal tip of the fibula (Fig. 2A, B, white arrow). At HH32, when the fibula is still roughly the same length as the tibia, the mitotic marker phosphohistone‐3 (PH3) reveals the presence of mitotic cells at both distal and proximal ends (Fig. 3A). However, by HH34, mitotic activity has been lost at its distal end, but not the proximal end, as the fibula becomes noticeably shorter than the tibia (Fig. 3B, C). These results demonstrate that by HH35 the distal end of the fibula is not comparable to a normal epiphysis: Rather, it shows early cartilage maturation and cessation of cell proliferation, comparable to what occurs in the diaphysis of other skeletal elements (Fig. 2C).

### PTHrP IS DOWNREGULATED IN THE DISTAL FIBULA, BUT STRONGLY EXPRESSED IN THE FIBULARE

**Figure 4:**
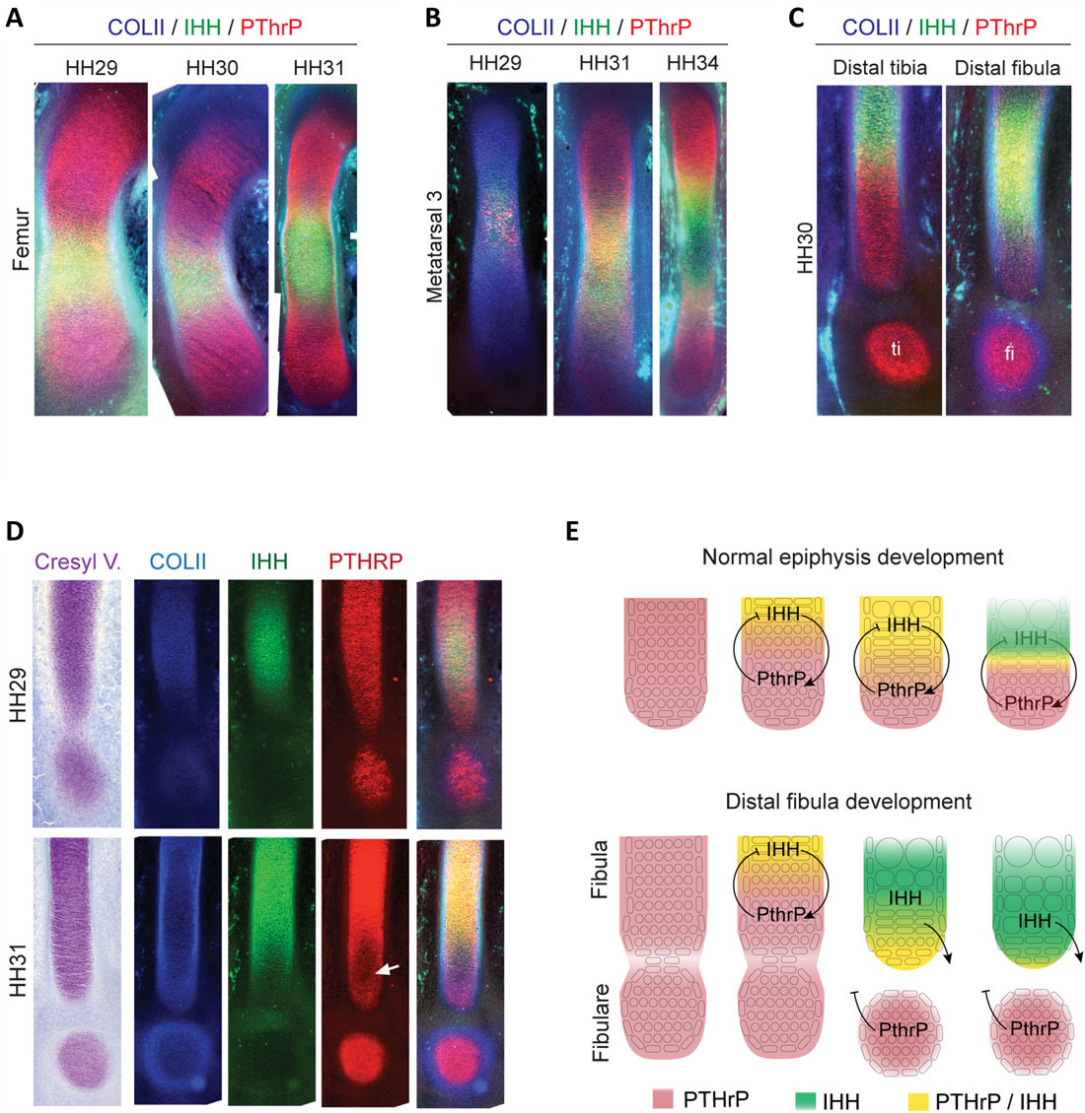
The fibulare interacts with the fibula during growth plate formation. Immunofluorescence against COLII (blue) IHH (green) and PTHrP (red) in the femur (A) and metatarsal 3 (B) show early coproduction of IHH and PTHrP. Later, PTHrP is displaced to the extremes of the cartilaginous element and IHH remains expressed in the center. Therafter, IHH ceases to be expressed at the center and becomes divided into two bands that progress as waves toward each end. (C) IHH expression is restricted to the center of the tibia and its distal end is not depleted from PTHrP, contrary to the fibula at the same stage. Yellow signal is coexpression of Ihh (green) and PTHrP (red). (D) Histological sections of the fibula of embryos in stages HH29 and HH31, stained with Cresyl Violet and triple immunofluorescence against Coll‐II, IHH, and PTHrP. The fibulare does not express IHH, but does express PTHrP, behaving as an epiphysis of the fibula during the time they remain in close contact. (E) Schematic representation of the production of IHH and PTHrP proteins in a “normal epiphysis” and in the distal fibula. The separation of the fibulare disrupts the feedback loop and causes the maturation of the distal fibula. ti: tibiale; fi: fibulare.

In growth plates, PTHrP is expressed at high levels in the periarticular region and at low levels in the underlying proliferating chondrocytes (Vortkamp et al. 1996; Kronenberg et al. 1997; St‐Jacques et al. 1999). We found that in chicken early embryonic cartilages, before the development of growth plates, PTHrP is produced throughout the entire leg skeleton. When IHH production begins in the center, it is initially coproduced with PTHrP (see the femur and metatarsal III in Fig. 4A, B). Only thereafter, PTHrP becomes downregulated at midshaft, and is absent within the IHH domain, generating two domains of pure PTHrP in the extremes, separated by a domain of pure IHH in the center. Early development of the fibula departs from this pattern: at HH29, when the fibulare is closely apressed to the fibula, the molecular profile of the distal end of the fibula resembles that of the midshaft of other elements, co‐expressing Ihh and PthrP. The fibulare in turn strongly expresses PTHrP, but no IHH, resembling the distal end of other elements (Fig. 4D, E). When the fibula is set apart from the fibulare at HH31, its epiphysis begins to mature in a diaphysis‐like fashion (Figs. 2C, 4E). Unlike any other element, PTHrP then becomes downregulated at the distal fibula (Fig. 4C, D, white arrow). IHH expression is initially low in this PTHrP‐free zone, but thereafter it progresses into the distal end of the fibula, reaching the periarticular region at HH35 (Figs. 2B, 4D). The abnormal molecular profile of the distal fibula reveals a failure of the distalmost epiphysis to establish a zone of strong PTHrP expression, suggesting a cause for its premature ossification (Fig. 4E).

### POSTMORPHOGENETIC DOWNREGULATION OF IHH SIGNALING LEADS TO EQUALLY LONG TIBIA AND FIBULA

**Figure 5:**
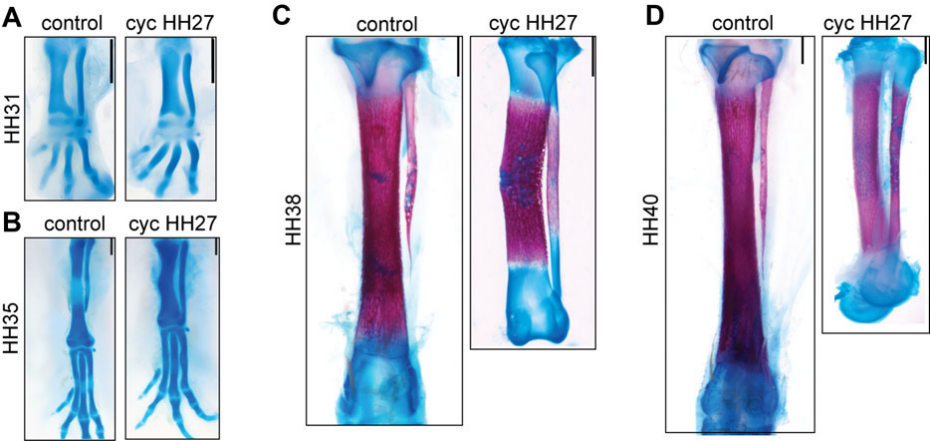
Experimental enlargement of the fibula depends on the fusion to the fibulare. Cyclopamine‐treated embryos have a larger fibula fused to the fibulare and conserve a cartilaginous distal end. The tibia is smaller and bent and presents a nodule or “bump” at midlength. HH31 (A) and HH36 (B) legs were stained with Alcian Blue; HH38 (C) and HH40 (D) legs were stained with Alcian Blue and Alizarin Red. Scale: 1 mm.

To assess the role of IHH signaling in the differentiation of the distal fibula, we treated chicken embryos *in ovo* with the hedgehog inhibitor Cyclopamine at a late, postmorphogenetic stage just before the onset of IHH production in the zeugopod (HH27, Rapacioli et al. 2012). This is long after the early role of Sonic Hedgehog (SHH) in patterning the early limb bud (Towers et al. 2008; Chinnaiya et al. 2014). At postmorphogenetic stages, IHH is the only member of the hedgehog family that is expressed at the limb skeleton (Kronenberg 2003; Kozhemyakina et al. 2015). Downregulation of IHH signal at HH27 with cyclopamine led to a fibula and tibia of similar length, and fusion of the fibula to the fibulare (Fig. 5). As in previously published experiments obtaining dinosaur‐like proportions, downregulation of IHH also leads to a significant decrease in growth of the tibia: the tibia was 9% and 21% smaller (1.54 ± 0.12 mm and 5.77 ± 0.38 mm) and the fibula became 3% and 6% larger (1.65 ± 0.17 mm and 4.37 ± 0.25 mm) than controls at HH31 and HH36, respectively (*n* = 10, Fig. 5A, B). Eight embryos were incubated until late stages (HH38 and HH40) had tibiae around two‐thirds the length of control embryos, slightly bent, developing a nodule or “bump” at midshaft. Fibulae total lengths were similar to control, but they had longer bone collars and their distal end remains cartilaginous. Its shape did not become splinter‐like, but remains tubular and dinosaur‐like (Fig. 5C). Whole mount immunofluorescence at HH31 shows the fused fibula‐fibulare has a molecular profile like that of a single element, with IHH expression at the center, whereas Coll‐II is expressed in the proximal epiphysis and the distal fibulare (Fig. S6).

### LOSS OF THE DISTAL EPIPHYSIS IN MESOZOIC STEM BIRDS PRECEDED THE EVOLUTION OF GREATER FIBULAR REDUCTION

**Figure 6:**
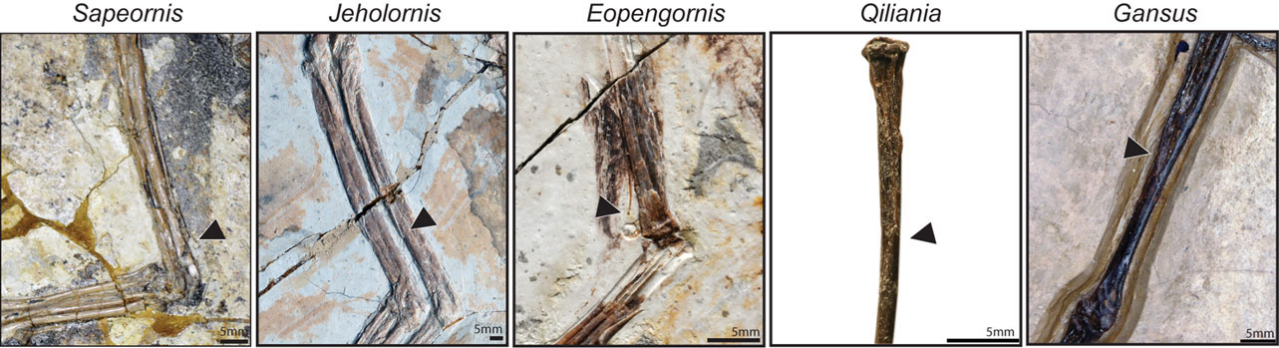
Fossil taxa documenting the early evolution of distal fibular reduction. Basal pygostylia such as *Sapeornis* and *Jeholornis*, and the basal enantiornithes *Eopengornis* show a splinter‐like distal fibula, lacking an epiphysis and articulation to the ankle. However, the fibula is not much shorter than the tibia. Thereafter, lower fibulo–tibial ratios evolved in different lineages, such as *Qiliania* within enantinornithes, and *Gansus* within Ornithotoraces.

**Figure 7:**
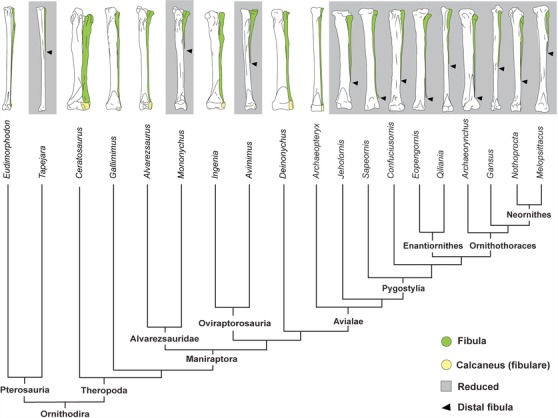
The convergent reduction of the distal fibula in Ornithodira. The ancestral condition for Ornithodira, clade that contains Pterosaurs and Dinosaurs, is a slender fibula with reduced space between it and the tibia. Thereafter, the fibula became distally reduced independently in four clades: Pterosauria, Alvarezsauridae, Oviraptorosauridae, and Avialae. Basal Mesozoic birds such as *Jeholornis, Sapeornis, Eopengornis*, and *Archaeorhynchus* had already evolved a splinter‐like distal fibula with no distal epiphysis, unarticulated to the ankle. However, the fibula remained only slightly shorter than the fibula. Lower fibulo–tibial ratios (as in *Confuciusornis, Quiliania*, and *Gansus*) evolved thereafter in independent lineages of birds.

The fossil record on the early evolution of birds has improved significantly in the last decades. With the exception of *Archaeopteryx*, all Mesozoic birds have a distally tapering, splinter‐like fibula that does not articulate with the ankle. Thus, formation of a normal distal epiphysis was lost early in avian evolution. However, the fibula was still almost as long as the tibia in basal Avialae such as *Sapeornis* (Zhou and Zhang 2003; Chongxi 2008; Gao et al. 2012; Pu et al. 2013); basal enantiornithes such as *Eopengornis*, *Pengornis*, and *Protopteryx* (Han et al. 2014); and basal ornithuromorphs such as *Archaeorhyncus* (Zhou et al. 2013, Figs. 6 and 7). Greater reduction of the fibula only evolved thereafter, having being independently acquired by Jeholornithiformes (Zhou and Zhang 2002; O'Connor et al. 2011; Lefèvre et al. 2014), Confuciusornithiformes (Chiappe et al. 1999; Zhang et al. 2009), derived Enantiornithes, and derived Ornithuromorpha, that are the ancestors of modern birds (O'Connor et al. 2011b; Zhou et al. 2013; Figs. 6 and 7). Thus, fossil evidence supports the evolution of a fibula that was splinter‐like, but almost as long as the tibia, before convergent evolution in different lineages of a strongly reduced fibula.

## Discussion

The hypothesis of mesenchymal competition suggests that interactions between the early precursor cell populations of the fibula and the tibia determine a “stunted” condensation of the fibula, and that these interactions may also explain variation among modern birds, some of which show greater fibular reduction than others (Hampe b; Streicher and Muller 1992). A minimum cell number is necessary to form a mesenchymal condensation, and experimental reductions in early limb bud cell number can cause the complete loss of the smaller element in the zeugopod (Wolff and Hampe 1954; Towers et al. 2008). However, the mechanisms that actually achieve fibular reduction in birds only come into effect long after the early patterning of condensations, at late, postmorphogenetic stages. In all bird species we observed, the embryonic fibula remains equal to the tibia near HH31, even when the fibula is drastically reduced, as in the budgerigar (Fig. S2). The hypothesis that the distal end of the fibula breaks off (Archer et al. 1983), although mistaken in structural terms, is correct in its functional implications, because the fibula fails to develop a distal growth plate: the undifferentiated population of cells at the distal fibula become terminally differentiated between HH31 and HH36. In contrast, proper epiphyseal growth plates are established at its proximal end, and at both ends of the tibia. As pointed out above, at HH29 of the chicken, the fibulare becomes closely appressed to the distal fibula. The molecular profile of the fibula suggests that, during this transient contact, the fibulare may act as a functional extension of the fibula or “surrogate epiphysis,” which may allow IHH expression to progress further into the actual distal end of the fibula. As the fibulare then separates from the fibula, the surrogate epiphysis is lost, but cartilage maturation still progresses, impairing the proper development of a growth plate at the distal fibula. This could explain why the bone collar of the fibula is off‐center and distally displaced during its early ossification in all bird species observed (Fig. S4).

Cyclopamine downregulates IHH signaling, and in treated legs it may delay the advance of cartilage maturation, as well as impairing the formation of an interzone between the fibula and the fibulare (Mak et al. 2006; Später et al. 2006; Gao et al. 2009; Ray et al. 2015). Fused to the fibulare, the fibula now retains its surrogate epiphysis. Importantly, the fibulare continues to express PTHrP, and no IHH, while maturation is well underway in the tibia and fibula. This is not surprising because in all tetrapods, tarsal elements ossify much later than other limb bones (Rieppel 1992; Caldwell 2002; Fröbisch 2008). The sustained expression of PTHrP at the fibulare may negatively regulate IHH, allowing the maintenance of immature chondrocytes and distal growth at the fibula until later stages. It is worth noting that in all experiments in which the fibula is as long as the tibia, the fibulare is fused to its distal end, despite radically different procedures (mesenchyme grafts, barrier insertions, *Shh*‐expressing cells grafts, Hox misexpression, and Cyclopamine treatment; Hampe 1958, 1960a,b; Archer et al. 1983; Müller 1989; Goff and Tabin 1997). Although different mechanisms somehow impede separation of fibulare and fibula, retention of the “surrogate epiphysis” may explain sustained growth of the fibula in all cases.

During evolution, in the advanced mesotarsal ankle of the basal ornithodira (the earliest bird ancestors to show upright, bipedal locomotion) the fibulare became closely locked into the fibula, with little or no movement between these bones. This greater adult proximity between proximal tarsals and the zeugopod is in fact reflected in early embryonic patterns: The tibiale and the fibulare of birds form very close to the tibia and fibula, in contrast with *Alligator* and other amniotes in which they are more separate, and movement of the foot involves rotation at their articulation (Fig. S3). The close embryonic proximity of the fibula and fibulare in avian embryos may have been common to all Ornithodira, allowing “surrogation” and loss of the distal fibular epiphysis in those clades in which differentiation of the fibula started before its separation from the fibulare. In fact, besides birds, distal fibular reduction also occurred independently within at least three other lineages of Ornithodira: Alvarezsauridae (Chiappe et al. 2002), Oviraptorosauria (Vickers‐Rich et al. 2002), and Pterosauria (Dalla Vecchia 2003; Bonaparte et al. 2010; Fig. 7).

It has been previously suggested that development of a smaller fibula occurs as a mere result of greater growth of the tibia (Müller 1989). Accordingly, it has been proposed that among modern birds, the evolution of a strongly reduced fibula has no adaptive value in itself, but is a developmental by‐product of selection for a longer tibia in specialized lifestyles such as wading (Streicher and Muller 1992). However, besides long‐legged waders (flamingos, storks, and herons), among modern birds the relatively smallest fibulae (less than 50% the length of the tibia) have also evolved in small, short‐legged birds (swifts, passerines, and kingfishers). The presence of strongly reduced fibulae in these short‐legged birds could be explained if small body size can also bring about greater fibular reduction (Streicher and Muller 1992). However, the alleged correlation between longer legs and reduced fibulae is also challenged by the fact that long‐legged birds such as cursorial ratites (ostriches, emus) and birds of prey (eagles, owls) display some of the relatively largest fibulae (more than 75% the length of the tibia; Baur 1885; Shufeldt 1894; Hampe b; Streicher and Muller 1992).

Our own embryological observations in birds with different degrees of fibula reduction show that roughly adult proportions are already attained in the specific period or “window” between stages HH31‐36, following failure of the fibula to maintain a distal growth plate. We infer this is when the tibia is most capable of outgrowing the fibula. Bird species with a greatly reduced fibula display greater growth of the tibia during this embryonic period, regardless of whether they are long‐ or short‐legged as adults. These embryological differences among bird species are hard to interpret in adaptive terms. Thus, although we agree that fibular reduction is probably in itself nonadaptive, it is not easy to identify another directly adaptive trait that leads to it as a by‐product. In developmental terms, our new data demonstrate that disruption of the fibular distal epiphysis occurs, which cannot be a mere side effect of greater growth at the tibia. Indeed, loss of the epiphysis may be required for greater growth of the tibia, such that, if the epiphysis is retained, growth in the tibia is significantly decreased.

In the context of our interpretation, we propose a two‐step hypothesis for the evolution of fibular reduction in birds. The earliest birds to show fibular reduction have a splinter‐like distal end that does not articulate the ankle, suggesting disruption of the growth plate. However, their fibula is still almost as long as the tibia (Fig. 7; Chiappe et al. 1999; Zhou and Zhang 2002; Wang et al. 2014). Increased reduction of the fibula only occurred thereafter and independently in several lineages of Mesozoic birds (including that leading to modern birds, Fig. 7) as an outcome of the evolution of greater growth of the tibia.

Previously, the loss of the distal fibular epiphysis was considered an alternative to greater embryonic growth of the tibia, as mutually exclusive hypotheses. In our view, greater growth in the tibia is important, but only during the embryonic period when the distal fibular epiphysis is prematurely ossifying. Like other recent work, our data stress the importance of postmorphogenetic changes for the origin of evolutionary novelties (Nagashima et al. 2009; Botelho et al. 2014b; Cooper et al. 2014), highlighting the role of spatial relations between skeletal elements at the time of the onset of endochondral ossification.

## Supporting information


**Figure S1**. Pharmacological inhibition of *Ihh* pathway.
**Figure S2**. The fibula is as large as the tibia at early development in species with relatively short fibulae.
**Figure S3**. The fibulare develops closer to the distal fibula in birds than in other extant reptiles.
**Figure S4**. Fibular reduction in species with different adult fibulo‐tibial ratios.
**Figure S5**. Fibular reduction depends on time and rate of differentiation.
**Figure S6**. Postmorphogenetic cyclopamine treatment causes the fusion of the fibulare to the fibula.
