# Supplementary material for: Molecular development of fibular reduction in birds and its evolution from dinosaurs
Source: Evolution. 2016 Mar 4;70(3):543–54. doi: 10.1111/evo.12882 (PMC5069580; doi:10.1111/evo.12882)
Supplement: Supplementary file 1 — Figure S1. Pharmacological inhibition of Ihh pathway. Figure S2. The fibula is as large as the tibia at early development in species with relatively short fibulae. Figure S3. The fibulare develops closer to the distal fibula in birds than in other extant reptiles. Figure S4. Fibular reduction in species with different adult fibulo‐tibial ratios. Figure S5. Fibular reduction depends on time and rate of differentiation. Figure S6. Postmorphogenetic cyclopamine treatment causes the fusion of the fibulare to the fibula. [file EVO-70-543-s001.pdf]

Molecular development of fibular reduction in birds and its evolution from dinosaurs

João Francisco Botelho, Daniel Smith-Paredes, Sergio Soto-Acuña, Jingmai O'Connor, Verónica Palma, Alexander Vargas

Supplementary Information

Table of Contents:

Supplementary Figure 1: Pharmacological inhibition of *Ihh* pathway

Supplementary Figure 2: The fibula is as large as the tibia at early development in species with relatively short fibulae

Supplementary Figure 3: The fibulare develops closer to the distal fibula in birds than in other extant reptiles

Supplementary Figure 4: Fibular reduction in species with different adult fibulo-tibial ratios

Supplementary Figure 5: Fibular reduction depends on time and rate of differentiation

Supplementary Figure 6: Post-morphogenetic Cyclopamine treatment causes the fusion of the fibulare to the fibula

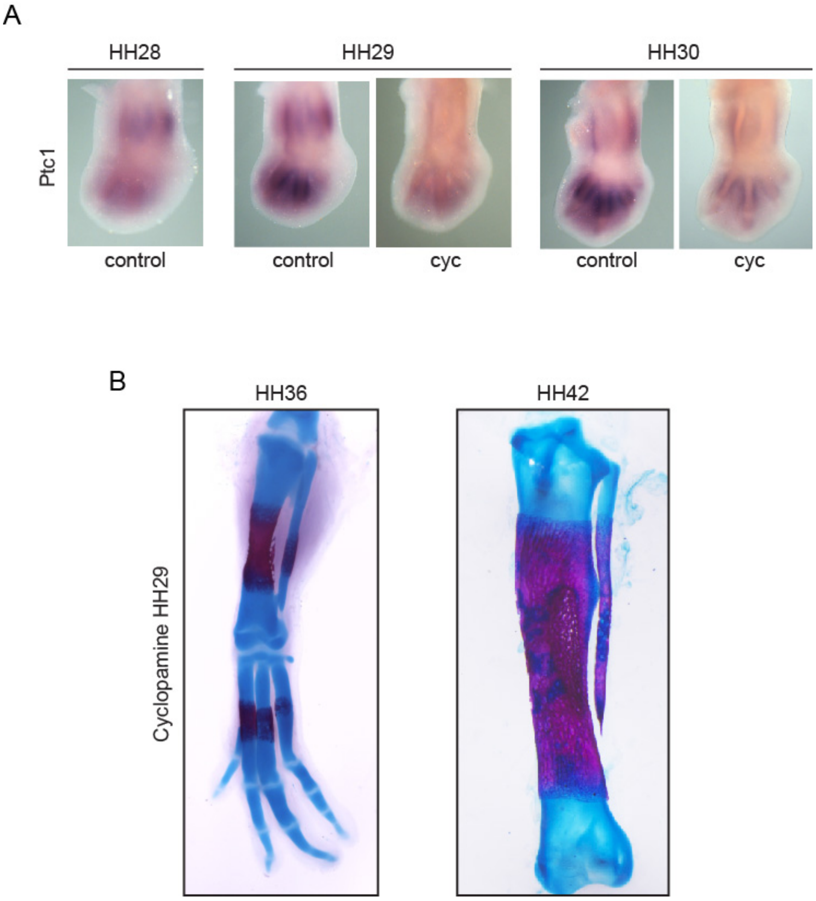

Supplementary Figure 1:

Pharmacological inhibition of *Ihh* pathway. (A) Late application of Cyclopamine disrupts *Ihh* signaling during endochondral ossification, as confirmed by the compared expression of one of its downstream targets, Ptc1, between control and experimental embryos; (B) Application of Cyclopamine at HH29 does not avoid distal fibular reduction in spite of reduced length of tibia and fibula.

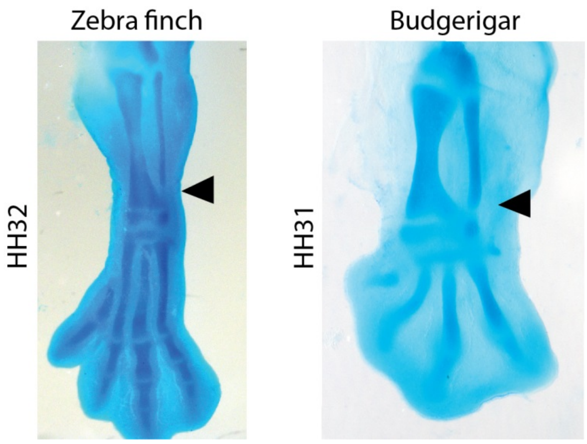

Supplementary Figure 2:

The fibula is as large as the tibia at early development in species with relatively short fibulae: Larger fibulae at early development of zebra finches and budgerigars indicate their reduction is ensuing at later stages.

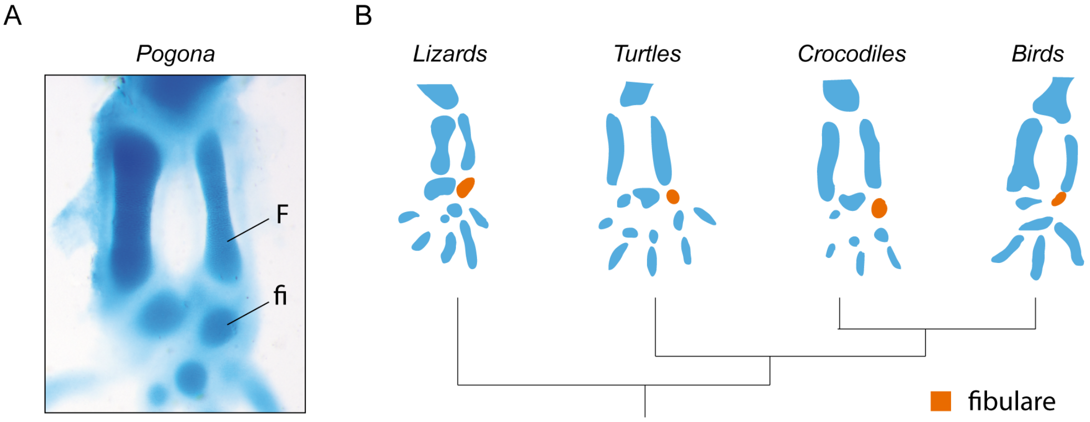

Supplementary Figure 3:

The fibulare develops closer to the distal fibula in birds than in other extant reptiles. (A) Bearded dragon (*Pogona vitticeps*) leg cartilages showing the fibula and the fibulare develop well separated; (B) Zeugopod development in other extant reptilia indicates that the proximity of the fibulare to the distal fibula at early development is a derived condition of birds (Drawings based on Müller and Alberch 1990; Rieppel 1993c; Rieppel 1993a; Rieppel 1993b; Rieppel 1994; Fabrezi et al. 2007).

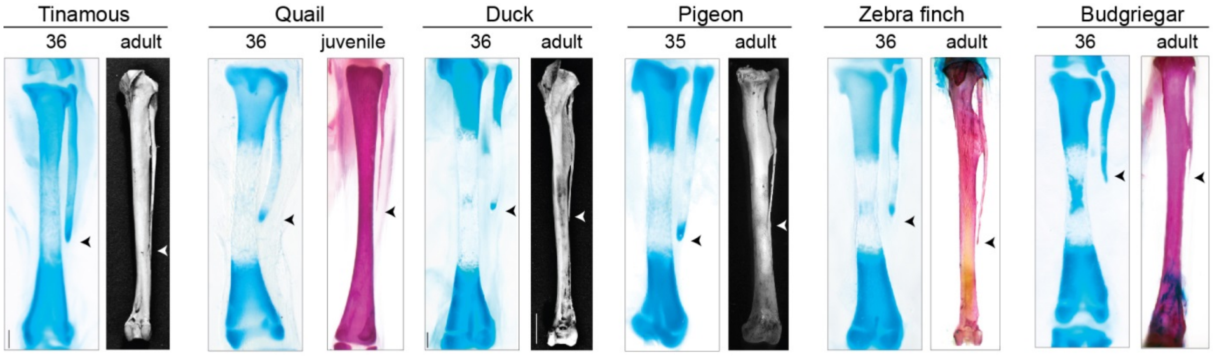

Supplementary Figure 4:

Fibular reduction in species with different adult fibulo-tibial ratios. In all species, essentially adult fibulo-tibial proportions have already been attained around stage HH36.

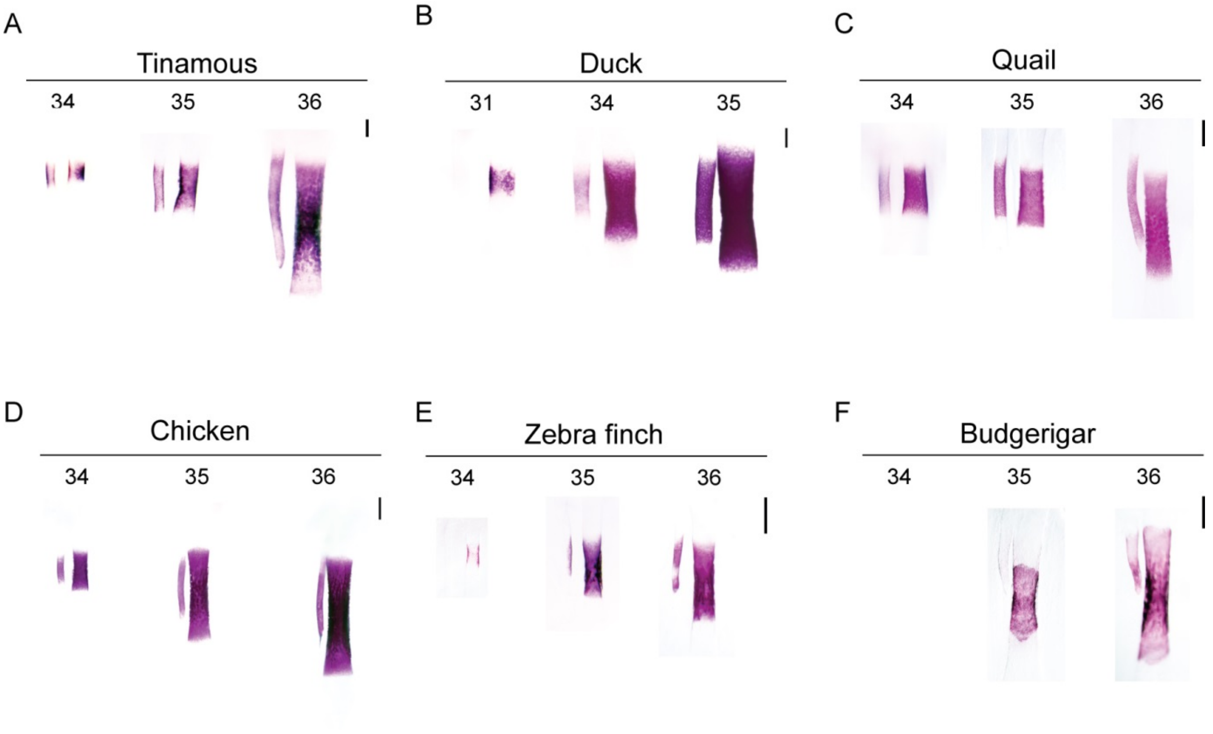

Supplementary Figure 5:

Fibular reduction depends on time and rate of differentiation. In species with lower fibulo-tibial ratios (duck, zebrafinch, budgerigar), ossification begins first in the tibia, and then in the fibula. Scale 300 µm.

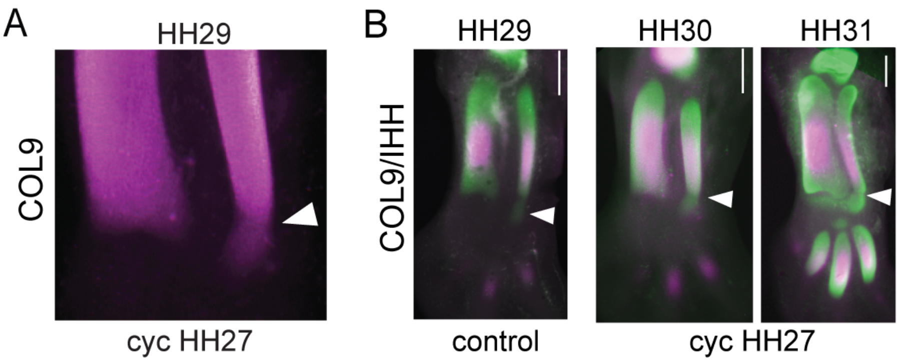

Supplementary Figure 6:

Post-morphogenetic Cyclopamine treatement causes the fusion of the fibulare to the fibula (A) Whole mount immunofluorescence showing the fusion of the fibulare to the distal fibula 24hr after Cyclopamine treatment (see figure 1B for comparison); (B) Whole mount double immunofluorescence shows that the fused fibula-fibulare has a molecular profile like that of a single element, with IHH expression at the center, while COL-II is expressed in the proximal epiphysis and the distal fibulare (white arrowheads indicate the fusion of fibulare to the fibula).

References

Fabrezi, M., V. Abdala, and M. I. M. Oliver. 2007. Developmental Basis of Limb Homology in Lizards. Anat. Rec., Part A 290:900-912.

Müller, G. B. and P. Alberch. 1990. Ontogeny of the limb skeleton in Alligator mississippiensis: developmental invariance and change in the evolution of archosaur limbs. J Morphol 203:151-164.

Rieppel, O. 1993a. Studies on Skeleton Formation in Reptiles .V. Patterns of Ossification in the Skeleton of Alligator-Mississippiensis Daudin (Reptilia, Crocodylia). Zool J Linn Soc 109:301-325.

Rieppel, O. 1993b. Studies on skeleton formation in reptiles: patterns of ossification in the skeleton of Chelydra serpentina (Reptilia, Testudines). Journal of Zoology 231:487-509.

Rieppel, O. 1993c. Studies on skeleton formation in reptiles. II. Chamaeleo hoehnelii (Squamata: Chamaeleoninae), with comments on the homology of carpal and tarsal bones. Herpetologica:66-78.

Rieppel, O. 1994. Studies on skeleton formation in reptiles. Patterns of ossification in the skeleton of Lacerta agilis exigua Eichwald (Reptilia, Squamata). J Herpetol:145-153.
